# Supplementary figures and images for: Glomerular developmental delay and proteinuria in the preterm neonatal rabbit
Source: PLoS One. 2020 Nov 9;15(11):e0241384. doi: 10.1371/journal.pone.0241384 (PMC7652305; doi:10.1371/journal.pone.0241384)

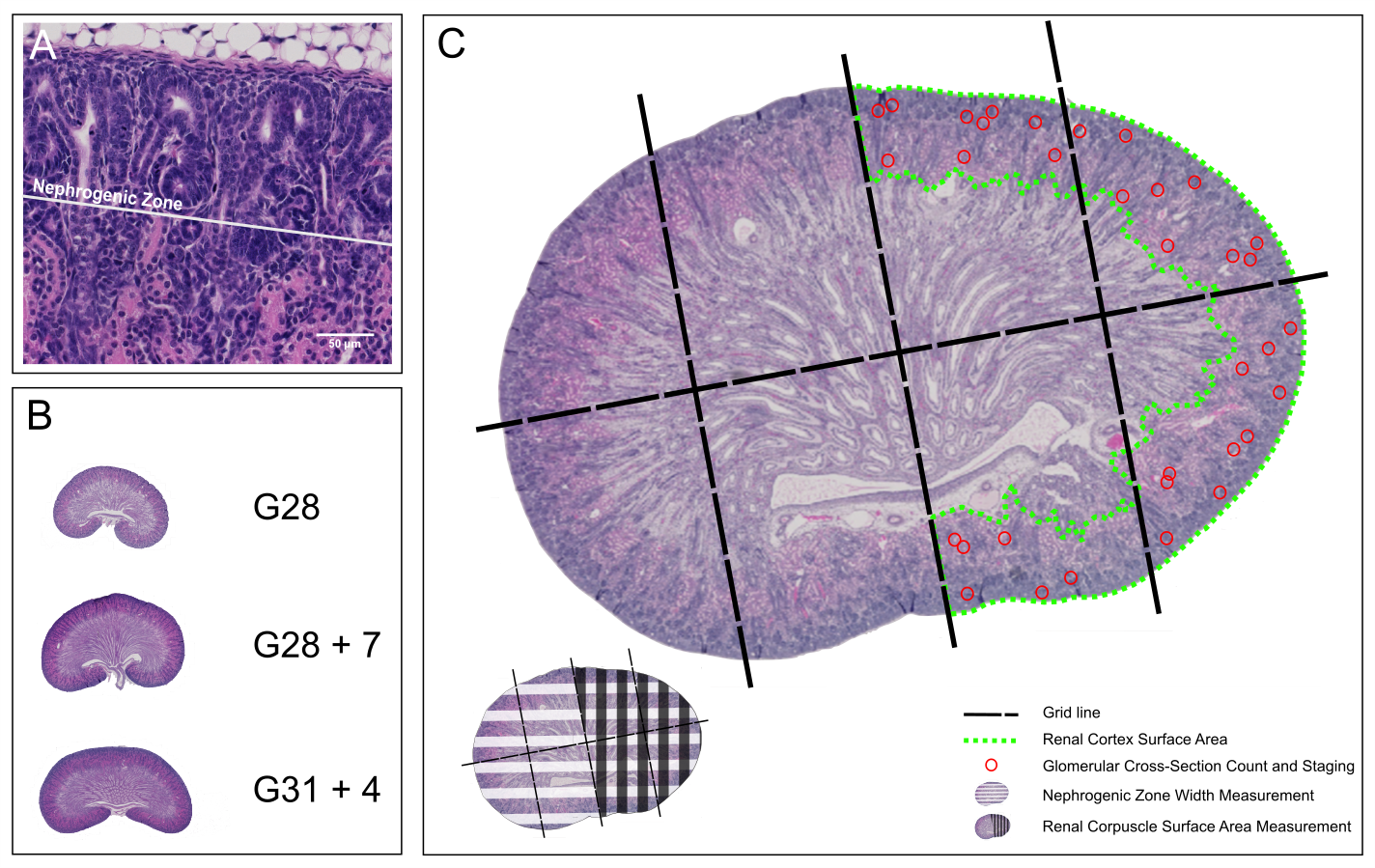

Supplement: S1 Fig — (A): Representative image of the nephrogenic zone as found in the outer renal cortex. The lower border of the nephrogenic zone is highlighted (white line). (B): Example histology slides for G28, G28 + 7 and G31 + 4. Provides a quick overview of the relative size. (C): Illustrative example of the analysis methodology. The kidney was divided in 8 sectors using grid lines. The upper or lower pole was randomly selected and all glomerular cross-sections were counted and staged. The renal cortex surface was measured by tracing just underneath the outer renal capsule and along the boundary between medulla and cortex. The nephrogenic zone width was measured in 8 sectors; the renal corpuscle surface area was measured in the 4 sectors in the randomly selected pole. (TIF) [file pone.0241384.s001.tif]
