# Supplementary material for: Glomerular developmental delay and proteinuria in the preterm neonatal rabbit
Source: PLoS One. 2020 Nov 9;15(11):e0241384. doi: 10.1371/journal.pone.0241384 (PMC7652305; doi:10.1371/journal.pone.0241384)
Supplement: S1 Table — G28, G28 + 7, and G31 + 4, presented as mean and 95% confidence intervals. (PDF) [file pone.0241384.s002.pdf]

	G28		G28 + 7		G31 + 4	
Total one-hour survival		-		6		6
Total N		8		5		6
Birthweight [g]	38.3	[35.0, 41.6]	39.9	[36.9, 42.9]	54.0	[42.4, 65.6]
Harvest weight [g]	38.3	[35.0, 41.6]	53.0*	[49.0, 57.0]	60.4**	[48.0, 72.8]
Kidney Volume [ml^3]	148.8	[127.1, 170.4]	362.0**	[305.5, 418.5]	381.7**	[297.9, 465.5]
Kidney Weight [mg]	151.9	[134.8, 169.0]	355.0**	[300.3, 409.7]	345.2**	[268.7, 421.7]
Relative Kidney Volume [ml^3/g]	3.9	[3.3, 4.5]	6.8**	[6.1, 7.5]	6.3**	[5.8, 6.9]
Relative Kidney Weight [mg/g]	4.0	[3.6, 4.3]	6.7**	[5.9, 7.5]	5.7**, †	[5.1, 6.3]
Nephrogenic Zone Width [µm]	179.9	[166.2, 193.5]	207.8	[178.6, 237.1]	229.2**	[206.9, 251.6]
Renal Cortex Surface Area [mm^2]	6.9	[6.4, 7.4]	11.3	[9.4, 13.1]	11.5	[10.1, 12.8]
Glomerular Density	27.6	[25.0, 30.2]	32.2	[28.4, 36.2]	31.5	[26.2, 36.8]
Renal Corpuscle Surface Area [inner cortex, μm^2]	3856.8	[3555.2, 4158.4]	2653.1**	[2347.7, 2958.5]	2459.8**	[2121.8, 2797.8]
Renal Corpuscle Surface Area [outer cortex, μm^2]	2302.8	[2083.3, 2522.3]	1332.0**	[1194.4, 1469.6]	1406.1**	[1246.0, 1566.2]

* $p < 0.05$ compared to G28
** $p < 0.001$ compared to G28
† $p < 0.05$ compared to G28 + 7
