# Supplementary material for: Glomerular developmental delay and proteinuria in the preterm neonatal rabbit
Source: PLoS One. 2020 Nov 9;15(11):e0241384. doi: 10.1371/journal.pone.0241384 (PMC7652305; doi:10.1371/journal.pone.0241384)
Supplement: Attachment — Submitted filename: Response to Reviewers.docx [file pone.0241384.s005.docx]

Dear Prof. Dr. Umberto Simeoni,

We appreciate the swift response and the helpful comments of the reviewer on our previously reviewed manuscript entitled “*Glomerular developmental delay and proteinuria in the preterm neonatal rabbit.”*

We have made the additional revisions based on the comments and suggestions of reviewer #2. These comments are stated below, followed by our response in bullet points and *Italics*.

Yours sincerely,

On behalf of the authors,

Derek de Winter

**Reviewer #2:**

1. “The small number of animals in each group likely resulted in type 2 error in the statistical analyses, and in a lack of significant differences where there may be biologic effects. This should be indicated in the discussion. The comment in the author's response to reviewers, "morphometric techniques that have been used in this experiment are sensitive to confounding" would be magnified by the small number of animals.”
   - *Indeed, the small number of animals in each experimental arm make it possible to create a type II statistical error, thus accepting a possibly false null hypothesis. We have adapted the manuscript in accordance and can be referenced on lines 355-357.*
2. “In the discussion, lines 344-348, the authors hypothesize that increased proteinuria may be caused by podocyte pathology or tubular backflow of creatinine, but do not mention reduced tubular protein reabsorption that was proposed by Tsukahara based on their beta-2 microglobulin data. This should be included.”
   - *The reviewer is right. Tsukahara et al. have evaluated the presence of albumin and beta-2 microglobulin in urine samples during the neonatal period. They have found that both albumin and beta-2 microglobulin were increasingly elevated with increasing degrees of prematurity. In fact, changes in beta-2 microglobulin were more noticeable in relation to gestational age. Thus, we have adapted the manuscript to include this explanation. The data and findings from Tsukahara et al. are described in lines 337-342.*
   - *In addition, we have adapted our hypothesis to fit the abovementioned findings by Tsukahara et al. The hypothesis is described from lines 344-349: “From this, we hypothesize that the increase in proteinuria in the absence of glomerular hypertrophy, found in preterm rabbits, may be caused by changes in the glomerular structure, such as podocyte immaturity or an “absolute” podocyte depletion (a decrease in the total number of healthy podocytes per glomerulus [29]) or through premature tubular processes such as tubular backflow of creatinine [28] and reduced tubular resorption [27].”*
