# Supplementary material for: Glomerular developmental delay and proteinuria in the preterm neonatal rabbit
Source: PLoS One. 2020 Nov 9;15(11):e0241384. doi: 10.1371/journal.pone.0241384 (PMC7652305; doi:10.1371/journal.pone.0241384)
Supplement: Attachment — Submitted filename: PONE-D-20-11602 reviewer comments.pdf [file pone.0241384.s003.pdf]

PLOS ONE

Glomerular developmental delay and proteinuria in the preterm neonatal rabbit.

--Manuscript Draft--

**Manuscript Number:**	PONE-D-20-11602
**Article Type:**	Research Article
**Full Title:**	Glomerular developmental delay and proteinuria in the preterm neonatal rabbit.
**Short Title:**	Prematurity and its effect on renal development and function
**Corresponding Author:**	Derek de Winter<br>Amsterdam Universitair Medische Centra<br>Amsterdam, NETHERLANDS
**Keywords:**	prematurity; preterm birth; nephrogenesis; renal disease
**Abstract:**	Recent advances in neonatal care have improved the survival rate of those born premature. But prenatal conditions, premature birth and clinical interventions can lead to transient and permanent problems in these fragile patients. Premature birth (<36 gestational weeks) occurs during critical renal development and maturation. Some consequences have been observed but the exact pathophysiology is still not entirely known. This experimental animal study aims to investigate the effect of premature birth on postnatal nephrogenesis in premature neonatal rabbits compared to term rabbits of the same corrected age. We analyzed renal morphology, glomerular maturity and functional parameters (proteinuria and protein/creatinine ratio) in three cohorts of rabbit pups: preterm (G28), preterm at day 7 of life (G28+7) and term at day 4 of life (G31+4). We found no significant differences in kidney volume and weight, and relative kidney volume between the cohorts. Nephrogenic zone width increased significantly over time when comparing G31 + 4 to G28. The renal corpuscle surface area, in the inner cortex and outer cortex, tended to decrease significantly after birth in both preterm and term groups. With regard to glomerular maturity, we found that the kidneys in the preterm cohorts were still in an immature state (presence of vesicles and capillary loop stage). Importantly, significant differences in proteinuria and protein/creatinine ratio were found. G28 + 7 showed increased proteinuria ($p = 0.019$) and an increased protein/creatinine ratio ($p = 0.023$) in comparison to G31 +4. In conclusion, these results suggest that the preterm rabbit kidney tends to linger in the immature glomerular stages and shows signs of a reduced renal functionality compared to the kidney born at term, which could in time lead to short- and long-term health consequences.
**Order of Authors:**	Derek de Winter
	Thomas Salaets
	André Gie
	Jan Deprest
	Elena Levtchenko
	Jaan Toelen
**Additional Information:**	
**Question**	**Response**
**Financial Disclosure**<br><br>Enter a financial disclosure statement that describes the sources of funding for the work included in this submission. Review the submission guidelines for detailed requirements. View published research articles from *PLOS ONE* for specific	AG is funded with support of the Erasmus+ Programme of the European Union (Framework Agreement number: 2013-0040). JD is funded by The Research Foundation – Flanders (FWO Flanders) as clinical researcher (1.801207). EL is funded by The Research Foundation – Flanders (FWO Flanders) as clinical investigator (1801110N). This study was supported by The Research Foundation – Flanders (FWO Flanders) (grant G0C4419N) and Katholieke Universiteit Leuven (C2 grant: C24/18/101). The funders had no role in study design, data collection and analysis, decision to publish, or preparation of the manuscript.

Powered by Editorial Manager® and ProduXion Manager® from Aries Systems Corporation

examples.<br><br>This statement is required for submission and **will appear in the published article** if the submission is accepted. Please make sure it is accurate.<br><br>**Unfunded studies**<br>Enter: *The author(s) received no specific funding for this work.*<br><br>**Funded studies**<br>Enter a statement with the following details:<br>• Initials of the authors who received each award<br>• Grant numbers awarded to each author<br>• The full name of each funder<br>• URL of each funder website<br>• Did the sponsors or funders play any role in the study design, data collection and analysis, decision to publish, or preparation of the manuscript?<br>• **NO** - Include this sentence at the end of your statement: *The funders had no role in study design, data collection and analysis, decision to publish, or preparation of the manuscript.*<br>• **YES** - Specify the role(s) played.<br><br>* typeset	
**Competing Interests**<br><br>Use the instructions below to enter a competing interest statement for this submission. On behalf of all authors, disclose any competing interests that could be perceived to bias this work—acknowledging all financial support and any other relevant financial or non-financial competing interests.<br><br>This statement **will appear in the published article** if the submission is accepted. Please make sure it is accurate. View published research articles from *PLOS ONE* for specific examples.	The authors have declared that no competing interests exist.

Powered by Editorial Manager® and ProduXion Manager® from Aries Systems Corporation

**NO authors have competing interests**<br><br>Enter: *The authors have declared that no competing interests exist.*<br><br>**Authors with competing interests**<br><br>Enter competing interest details beginning with this statement:<br><br>*I have read the journal's policy and the authors of this manuscript have the following competing interests: [insert competing interests here]*<br><br>* typeset	
**Ethics Statement**<br><br>Enter an ethics statement for this submission. This statement is required if the study involved:<br><br>• Human participants<br>• Human specimens or tissue<br>• Vertebrate animals or cephalopods<br>• Vertebrate embryos or tissues<br>• Field research<br><br>Write "N/A" if the submission does not require an ethics statement.<br><br>General guidance is provided below. Consult the submission guidelines for detailed instructions. **Make sure that all information entered here is included in the Methods section of the manuscript.**	All experiments were approved by the Ethics Committee for Animal Experimentation (P060/2016 and P080/2017) and were performed according to local guidelines on animal welfare. Does were adequately sedated and then euthanized by administrating an intravenous injection of a mixture of 200 mg embutramide, 50 mg mebezonium, and 5 mg tetracain hydrochloride. Pups were deeply sedated with ketamine 35 mg/kg bodyweight (Nimatek®; Eurovet Animal Health BV, Bladel, The Netherlands) and xylazin 6 mg/kg bodyweight (XYL-M®; VMD, Arendonk, Belgium). After adequate sedation, the abdomen was opened through laparotomy and pups were euthanized by exsanguination. Fetal controls were prevented from breathing by not removing the fetal membranes.

**Format for specific study types**<br><br>**Human Subject Research (involving human participants and/or tissue)**<br>• Give the name of the institutional review board or ethics committee that approved the study<br>• Include the approval number and/or a statement indicating approval of this research<br>• Indicate the form of consent obtained (written/oral) or the reason that consent was not obtained (e.g. the data were analyzed anonymously)<br><br>**Animal Research (involving vertebrate animals, embryos or tissues)**<br>• Provide the name of the Institutional Animal Care and Use Committee (IACUC) or other relevant ethics board that reviewed the study protocol, and indicate whether they approved this research or granted a formal waiver of ethical approval<br>• Include an approval number if one was obtained<br>• If the study involved *non-human primates*, add *additional details* about animal welfare and steps taken to ameliorate suffering<br>• If anesthesia, euthanasia, or any kind of animal sacrifice is part of the study, include briefly which substances and/or methods were applied<br><br>**Field Research**<br><br>Include the following details if this study involves the collection of plant, animal, or other materials from a natural setting:<br>• Field permit number<br>• Name of the institution or relevant body that granted permission	
**Data Availability**<br><br>Authors are required to make all data underlying the findings described fully available, without restriction, and from the time of publication. PLOS allows rare exceptions to address legal and ethical concerns. See the PLOS Data Policy and FAQ for detailed information.	Yes - all data are fully available without restriction

A Data Availability Statement describing where the data can be found is required at submission. Your answers to this question constitute the Data Availability Statement and **will be published in the article**, if accepted.<br><br>**Important:** Stating 'data available on request from the author' is not sufficient. If your data are only available upon request, select 'No' for the first question and explain your exceptional situation in the text box.<br><br>Do the authors confirm that all data underlying the findings described in their manuscript are fully available without restriction?	
**Describe where the data may be found in full sentences. If you are copying our sample text, replace any instances of XXX with the appropriate details.**<br><br>• If the data are **held or will be held in a public repository**, include URLs, accession numbers or DOIs. If this information will only be available after acceptance, indicate this by ticking the box below. For example: *All XXX files are available from the XXX database (accession number(s) XXX, XXX.*).<br>• If the data are all contained **within the manuscript and/or Supporting Information files**, enter the following: *All relevant data are within the manuscript and its Supporting Information files.*<br>• If neither of these applies but you are able to provide **details of access elsewhere**, with or without limitations, please do so. For example:<br><br>*Data cannot be shared publicly because of [XXX]. Data are available from the XXX Institutional Data Access / Ethics Committee (contact via XXX) for researchers who meet the criteria for access to confidential data.*<br><br>*The data underlying the results presented in the study are available from (include the name of the third party*	All raw data is available from the Zenodo database (DOI: 10.5281/zenodo.3760389)

and contact information or URL).<br>• This text is appropriate if the data are owned by a third party and authors do not have permission to share the data.<br><br>* typeset	
Additional data availability information:	Tick here if the URLs/accession numbers/DOIs will be available only after acceptance of the manuscript for publication so that we can ensure their inclusion before publication.

Powered by Editorial Manager® and ProduXion Manager® from Aries Systems Corporation

Manuscript
Click here to access/download;Manuscript;Manuscript_DdeWinter

Glomerular developmental delay and proteinuria in the preterm neonatal rabbit.

Derek de Winter[1*], Thomas Salaets[2,3], André Gie[2], Jan Deprest[2,4,5], Elena Levtchenko[2,3], Jaan Toelen[2,3]

1. Faculty of Medicine, Amsterdam UMC, The Netherlands
2. Department of Development and Regeneration, KU Leuven, Leuven, Belgium
3. Department of Paediatrics, Division Woman and Child, University Hospitals Leuven, Leuven, Belgium
4. Department of Obstetrics and Gynaecology, Division Woman and Child, University Hospitals Leuven, Leuven, Belgium
5. Institute for Women's Health, University College London, London, UK

* Corresponding author
E-mail: derekdwinter@gmail.com (DDW)

Abstract

Recent advances in neonatal care have improved the survival rate of those born premature. But prenatal conditions, premature birth and clinical interventions can lead to transient and permanent problems in these fragile patients. Premature birth (<36 gestational weeks) occurs during critical renal development and maturation. Some consequences have been observed but the exact pathophysiology is still not entirely known. This experimental animal study aims to investigate the effect of premature birth on postnatal nephrogenesis in premature neonatal rabbits compared to term rabbits of the same corrected age. We analyzed renal morphology, glomerular maturity and functional parameters (proteinuria and protein/creatinine ratio) in three cohorts of rabbit pups: preterm (G28), preterm at day 7 of life (G28+7) and term at day 4 of life (G31+4). We found no significant differences in kidney volume and weight, and relative kidney volume between the cohorts. Nephrogenic zone width increased significantly over time when comparing G31 + 4 to G28. The renal corpuscle surface area, in the inner cortex and outer cortex, tended to decrease significantly after birth in both preterm and term groups. With regard to glomerular maturity, we found that the kidneys in the preterm cohorts were still in an immature state (presence of vesicles and capillary loop stage). Importantly, significant differences in proteinuria and protein/creatinine ratio were found. G28 + 7 showed increased proteinuria ($p = 0.019$) and an increased protein/creatinine ratio ($p = 0.023$) in comparison to G31 +4. In conclusion, these results suggest that the preterm rabbit kidney tends to linger in the immature glomerular stages and shows signs of a reduced renal functionality compared to the kidney born at term, which could in time lead to short- and long-term health consequences.

Introduction

Recent advances in neonatal care have allowed the survival of extremely premature infants. At present more than 95% of infants born preterm survive into adulthood. The prenatal conditions that cause preterm labor, the insult of preterm birth itself and the interventions

during the clinical management of these fragile patients can all lead to transient and permanent effects on organ structure and function. This is often more evident for the pulmonary and nervous system compared to other organs, such as the kidneys.

Premature birth (<36 gestational weeks) affects the normal development and maturation of the renal system during a critical period. Nephrogenesis in humans is completed in week 34-36 of gestation, with a rapid formation of new nephrons from week 20 of gestation [1]. Preterm birth arrests this process and can result in a lower nephron number which does not recover later in life. Preterm birth itself is associated with neonatal acute kidney injury due to possible insults like decreased kidney perfusion (patent ductus, resuscitation, sepsis) or nephrotoxic medications. The resulting kidney damage is associated with renal pathology later in life such as hypertension, proteinuria and a decreased glomerular filtration rate [2-9].

Animal models are needed to study the exact pathogenesis or underlying mechanisms of kidney injury and disease caused by premature birth. Of the available models, mice and rats are most often used to study nephropathy, usually induced by surgical operation, drugs or toxins, or genetic modification. For neonatal pathology the rat seems an excellent model as active nephrogenesis persists until day 10 after birth. However, the rabbit model holds a position between the small and larger animal models and could thus provide a unique position for the translation of research to the clinic [10].

In this study we investigated the effect of preterm birth on nephrogenesis in preterm neonatal rabbits compared to term rabbits of the same corrected age.

Materials and methods

Ethical approval

The experiment was approved by the Ethics committee for Animal Experimentation of the Faculty of Medicine (KU Leuven; p060/2016) and performed according to current animal welfare guidelines and recommendations. All researchers involved in animal experimentation were trained and certified in accordance with the Federation of European Laboratory Animal Science Associations (FELASA) standards. Time-mated pregnant does (*New Zealand White and Dendermonde hybrid rabbits*) were provided by the animalium of the KU Leuven and housed in separate cages until caesarian section at a gestational age of 28 days (preterm) or 31 days (term). The cages were set at a 12 hour light-dark cycle, room temperature and free access to water and pellet feed.

Experimental procedure

Prior to caesarian section, does were sedated with intramuscular ketamine 35 mg/kg bodyweight (Nimatek®; Eurovet Animal Health BV, Bladel, The Netherlands) and xylazin 6 mg/kg bodyweight (XYL-M®; VMD, Arendonk, Belgium). Does were placed in a supine position after adequate sedation and euthanized by administrating an intravenous injection of a mixture of 200 mg embutramide, 50 mg mebezonium, and 5 mg tetracain hydrochloride (T61®; Intervet International BV, Boxmeer, The Netherlands). Then, the uterus was rapidly exposed by creating an abdominal incision and all pups were delivered through hysterotomy.

From each mother allocated to preterm delivery (G28), 2-3 fetuses were randomly selected for fetal harvest. These fetal controls were prevented from breathing by not removing the fetal membranes. The remaining pups were dried, stimulated and put in an incubator at 32 °C, 75% humidity, and 95% O_2.

Pups were weighed and labeled. From then onwards, pups were fed twice daily through a 3.5 Fr orogastric tube with milk replacer (Day One®, Protein 30%, Fat 50%; FoxValley; IL, USA) prepared according to manufacturer's guidelines. The quantity of feeding increased per

postnatal day (PN) from 40 (PN day 0), to 50 (PN day 1), to 75 (PN day 2), and finally 100 mg/kg bodyweight on PN day 3 until harvest. Immunoglobins (Col-o-Cat®, SanoBest; Hertogenbosch, The Netherlands) were added during the first 2 PN days and probiotics, electrolytes and vitamins (Bio-Lapis®; Probiotics International Ltd.; Somerser, UK) were added during the first 5 PN days. A single intramuscular injection of vitamin K1 was administered on PN day 2 (0.002 mg/kg BW, Konakion pediatrique®; Roche, Basel, Switzerland). From PN day 2 onwards pups were given daily intramuscular injections of benzylpenicillin (20,000 I.U./kg BW Penicilline®; Kela, Sint-Niklaas, Belgium) and amikacin (20 mg/kg BW day, Amukin®; Bristol-Myers-Squibb, Brussels, Belgium). Animals were checked twice daily during the regular feeding and weighing moments at 8 AM and 5 PM. Animals that showed signs of preterminal suffering, for example gasping, overstretching, laying in a supine position, were excluded from the experiment and euthanized as soon as possible by a single overdose intramuscular injection of ketamine. Immediate application of humane endpoints was not achievable for animals that died overnight during unsupervised moments.

Tissue collecting and processing

Preterm pups allocated to the G28 + 7 cohort were euthanized on postnatal day 7, whilst term pups allocated to the G31 + 4 cohort were euthanized on postnatal day 4. Pups were deeply sedated with ketamine 35 mg/kg bodyweight (Nimatek®; Eurovet Animal Health BV, Bladel, The Netherlands) and xylazin 6 mg/kg bodyweight (XYL-M®; VMD, Arendonk, Belgium). After adequate sedation, the abdomen was opened through laparotomy and pups were euthanized by exsanguination. Blood from G28 + 7 and G31 + 4 pups was aspirated from the right ventricle, heparinized and centrifuged to retrieve plasma. Urine from G28 + 7 and G31 + 4 pups was aspirated directly from the bladder. Then, left kidneys were harvested and the perinephric fat was removed. Kidneys were weighed and fixated in PFA 4% for 24 hours. Then, kidney volume was measured by water displacement and the fixated kidneys

were transferred to ethanol 70% for storage. Relative kidney volume and weight were calculated by dividing the kidney volume and weight by bodyweight at harvest.

One complete paraffin section at a thickness of 5 µm through the mid-hilar region on the longitudinal axis was collected for each kidney and stained with hematoxylin and eosin (H&E). Sections were digitalized using a Carl Zeiss Axio Scan.Z1 scanner at 40x magnification (0,220 μm^2 per pixel) and assessed blinded, by coding of the file names. Measurements were done by a single researcher using QuPath for Windows, Version 0.1.2 [11]. Unblinding was done after finishing measurements in all histology slides.

Histological assessment

Kidney slides were digitally divided in equal poles (based on surface area) of which one pole was randomly selected (for more details see S1 Fig.).

The distribution of maturity was quantified by counting and scoring all identifiable glomerular cross-sections in the selected half. Glomerular cross-sections unidentifiable for their maturity stage were excluded. Glomerular maturity stages were scored according to parameters described in Fig 1. Each stage was then averaged and calculated as the percentage of the total glomerular cross-sections counted.

Fig 1. Glomerular maturity stages found in the neonatal rabbit kidney. Stages are based on criteria previously defined [12-14]. Glomeruli were divided in either immature stages (A-C) or mature stages (D-F). **(A)** Vesicle (V): a cluster of mesenchymal cells, exclusively found in the nephrogenic zone, bordering a ureteric bud (UB). **(B)** Comma- or S-shaped body: formed by the elongation and twisting of the vesicle. **(C)** Capillary loop: The lower limb of the S-shaped body formed an immature glomerulus, characterized by a crescent shape and few capillaries. **(D)** Stage I: no lobulation and at least half of the

circumference is lined with dark-staining podocytes. **(E)** Stage II: little lobulation and less than half of the circumference is covered with dark-staining podocytes. Some open capillaries may be seen. **(C)** Stage III: fully matured glomerulus showing full lobulation and open capillaries. No podocytes lining the glomerular tuft. (*Bar = 50 µm*).

Glomerular density - the number of glomerular cross-sections per surface area - was calculated by dividing the total glomerular cross-sections by the renal cortex surface area in mm^2. The renal cortex surface area was defined as the area between the medulla and the fibrous renal capsule and was measured by tracing these borders.

Nephrogenic zone width [µm] was defined as the area in the outer renal cortex just below the fibrous renal capsule to the last appearance of comma- and S-shaped bodies (S1 Fig) [15]. Histology slides were divided in eight equal sectors in which one measurement per sector, following the major organ surface area, was performed. Finally, the average nephrogenic zone width was calculated for each slide.

Renal corpuscle cross-sectional surface area [μm^2] was measured by tracing the inner lining of the Bowman's capsule. All glomerular cross-sections (stages I-III for the inner cortex, and capillary loops for the outer cortex) were numbered. For both the inner and outer cortex 30 glomerular cross-sections or the maximum amount possible, were randomly selected using a random number generator. Then, the average renal corpuscle cross-sectional surface area for the inner and outer cortex was calculated.

Functional parameters

Urinary creatinine [mg/dL] was determined through Roche enzymatic method, and urinary protein [g/L] with the benzethonium chloride method [16]. From this the protein/creatinine ratio was determined [g/g creatinine]. Plasma creatinine concentration [mg/dL] was

determined by colorimetric assay (modified Jaffe reaction) [17]. Functional parameter data is presented as mean [CI-95%].

Statistical analyses

Statistical analyses were performed using GraphPad Prism version 7.04 for Windows (GraphPad Software, La Jolla California USA, www.graphpad.com). A p value < 0.05 was considered statistically significant. All variables were assessed for normality using the Shapiro-Wilk test. Bodyweight, absolute and relative kidney volume and kidney weight, glomerular density, nephrogenic zone width, renal corpuscle cross-sectional surface area and the distribution of maturity were tested with a one-way analysis of variance (ANOVA) and a post hoc Tukey's multiple comparisons test. Functional data was tested with an independent samples T-test including Levene's test for equality of variances. Correlations on continuous variables were tested using the Spearman's Rank-Order correlation. Best-fit linear lines were plotted using linear regression analysis.

Results

Animal survival and biometry

A total of six mothers were used in this experiment of which four were randomly allocated to the fetal preterm (G28) and preterm until postnatal day 7 (G28 + 7) condition and two were allocated for term delivery. This resulted in a total of 19 pups that were used in this experiment (S1 Table). Eight G28 pups were euthanized directly after birth and six G28 pups were raised until PN day 7 (G28 + 7). Six term pups were raised until PN day 4 (G31 + 4). One pup of the G28 + 7 cohort died overnight of unknown causes and was not included in the final analysis. This finally resulted in 8 G28, 5 G28 + 7 and 6 G31 + 4 pups. Birthweights of G28 and G28 + 7 were comparable. Even though harvest weight yielded no significant

difference between G28 + 7 and G31 + 4, bodyweight tended to be higher in the term group ($p = 0.245$).

Kidney morphology

Relative kidney weight was found to be higher in G28 + 7 compared to G31 + 4 ($p = 0.023$) (Fig 2). We found no significant differences in absolute kidney volume and weight, and relative kidney volume between G28 + 7 and G31 + 4, but in both term and preterm it tended to increase compared to fetal controls. Moreover, we found that the average nephrogenic zone width increased overtime when comparing G31 + 4 to G28 ($p < 0.001$). But, this effect was not found when comparing G28 + 7 to G28. Nevertheless, the nephrogenic zone width was not significantly different between G28 + 7 and G31 + 4. The total glomerular cross-sectional count (Fig 3), renal cortex surface area (S1 Table) and glomerular density (Fig. 2) tended to increase compared to fetal controls, but were comparable between G28 + 7 and G31 + 4. Post hoc Tukey's revealed no significant differences in renal corpuscle surface area between G28 + 7 and G31 + 4 (S1 Table). The renal corpuscle surface area, in the inner cortex and outer cortex, tended to decrease significantly ($p < 0.001$) after birth in both preterm and term groups. In addition, we found a negative correlation between the renal corpuscle surface area – for both the inner and outer cortex – and the glomerular cross-sectional count (Fig 3). Moreover, we found a positive correlation between the glomerular cross-sectional count and bodyweight ($r = 0.7931$), kidney volume ($r = 0.8811$) and kidney weight ($r = 0.8957$), indicating that the larger kidney cross-sections have more glomerular cross-sections.

Fig 2. (A): Visual illustration of the animal model. Time points highlighted in blue are harvesting time points. (B): A graphic overview of the total immature and mature stage glomeruli across G28, G28 + 7 and G31 + 4, in absolute [mean] and relative counts [mean / %].

Fig 3. (A) Glomerular cross-sections counted. (** = p < 0.001. Error bars: mean ± SD) (B-F) Correlations plotted using Spearman's Rank-Order correlation on glomerular cross-sections and the following variables: bodyweight, kidney volume, kidney weight, and renal corpuscle surface area for the inner and outer cortex. Best-fit slope (solid line) is accompanied by a 95%-CI (dotted line).

Glomerular maturity

Immature stages, taken overall, were found to be dominant in each group (Fig 2B and Table 1), specifically, vesicles in G28 (27.3%) and capillary loops in G28 + 7 (27.6%). Even though immature stages were dominant in G31 +4, we mostly found stage I glomeruli (31.0%). In comparison to fetal controls both G28 + 7 and G31 + 4 tended to have an increased absolute count of immature stages and stage I and II glomeruli. We found that G28 + 7 tended to have a higher relative count of overall immature glomerular stages than G31 + 4 (p = 0.136), and vice versa. Furthermore, post hoc Tukey's yielded no significant differences between G28 + 7 and G31 + 4 in vesicles (p = 0.064) and stage I glomeruli (p = 0.057). No significant differences were found in the relative counts of comma or S-shaped bodies, capillary loops, stage II or stage III glomeruli.

Table 1: Distribution of maturity stages. Stages presented by the absolute mean, 95% confidence interval[illegible] glomerular cross-sections counted.

**Stage**	**G28**			**G28 + 7**		
	Absolute	95%-CI	Relative	Absolute	95%-CI	Re[illegible]
**Immature Stages**	122.9	[96.9, 148.9]	65.5%	241.0**	[198.0, 284.0]	6[illegible]
Vesicle	52.8	[41.8, 63.8]	27.3%	79.2*	[64.5, 93.9]	21[illegible]
Comma or S-shaped Body	33.5	[29.0, 38.0]	17.6%	61.8**	[52.6, 71.0]	1[illegible]
Capillary Loop	40.1	[28.1, 52.1]	20.6%	100.0**	[69.8, 130.2]	2[illegible]
**Mature Stages**	70.0	[62.0, 78.0]	34.5%	121.2*	[97.1, 145.3]	3[illegible]
Stage I	45.8	[38.3, 53.3]	24.0%	88.6*	[68.2, 109.0]	2[illegible]
Stage II	16.4	[12.6, 20.2]	8.8%	29.4	[26.5, 32.3]	8[illegible]
Stage III	3.1	[1.3, 5.0]	1.7%	3.2	[0.5, 5.9]	0[illegible]

* $p < 0.05$ compared to G28
** $p < 0.001$ compared to G28

Functional parameters

We found a significant difference in urinary protein ($p = 0.019$) and protein/creatinine ratio ($p = 0.023$) between G28 + 7 and G31 +4 (Fig 4). No differences were found in urinary creatinine nor in plasma creatinine.

Fig 4. Functional parameters. Visual representation of the functional parameters measured: urinary creatinine, protein and the protein/creatinine ratio. Comparison between

G28 + 7 and G31 + 4 reveals significant differences in proteinuria and in the protein/creatinine ratio. (* = $p < 0.05$. Error bars: mean ± SD).

Discussion

In humans preterm birth exacts a toll on many organ systems, especially the lung, brain and kidney. This results in an increased morbidity later in life with obstructive lung pathology, impaired neurocognitive outcomes and chronic renal disease with hypertension [2-9], but the pathophysiological background is still not entirely known. This is in part due to the lack of data from human subjects, because of ethical concerns and a large developmental variability [18], which forces researchers to rely on animal studies. In recent years we have developed a rabbit model to study the changes in pulmonary development that are induced by prematurity and hyperoxia. In the case of lung development, the rabbit mimics the human neonatal situation quite well as rabbits also reach the alveolar stage before birth. Also for the central nervous system we found that the rabbit was a good proxy for humans as they develop a form of encephalopathy of prematurity [19]. In order to further explore the translational value of the preterm rabbit model we set out to study the effect on renal development.

We evaluated the effect of preterm birth on nephrogenesis in preterm neonatal rabbits compared to term rabbits of the same corrected age. For this we established three cohorts: G28, G28 + 7 and G31 + 4. This allowed us to study the effect of 3 additional intrauterine days by comparing G28 + 7 (28 intrauterine - and 7 extrauterine days) and gestational corrected G31 + 4 (31 intrauterine – and 4 extrauterine days) to the ‘fetal preterm stage’ at a gestation of 28 days. To our knowledge this is the first study to study the effects of prematurity on glomerular maturation and functionality.

When we analyzed the major structural aspects of the renal structures, we found that the kidney-to-bodyweight ratio was significantly higher in preterm rabbit pups (G28), which is in line with studies performed in human and non-human primate neonates [14,20,21]. But the difference of three days of intrauterine life did not result in major structural discrepancies between the two cohorts. The kidney-volume-to-bodyweight ratio and glomerular density did not differ between the G28 + 7 and G31 + 4 cohorts.

Yet the glomerular density as such may not be a sensitive enough marker to quantify differences between these two conditions. We performed a detailed assessment of the different stages of glomerular development and found out that the preterm rabbit kidney at a postnatal age of 7 days (G28 + 7) contains more immature glomerular structures (vesicles) compared with 'term' kidneys at a similar age (G31 + 4), which contain more stage I glomeruli. This suggests a significant disruption of normal nephrogenesis induced by prematurity. One can only speculate on the exact pathophysiological processes that would result in an altered nephrogenesis after birth in our model (e.g. water- and electrolyte disturbances, toxic effects of medication…).

In contrast to our findings, Sutherland et al. [14] found an accelerated maturation, with a decreased relative amount of vesicles and a smaller nephrogenic zone width in autopsied preterm human neonates whose post-natal survival ranged from 2 to 68 days, compared to still-born gestational controls. However, it remains unclear whether this difference was found because of the effect of prematurity or through the administration of antenatal steroids. Additionally, a study comparing preterm and term baboons found an interruption of normal kidney development, objectified through a decrease in glomerular generations and glomerular relative area with an increase of the renal corpuscle area [22].

It has been reported that nephrogenesis in the rabbit is completed in the 2nd or 3rd postnatal week [23]. In this respect the rabbit differs significantly from humans where the formation of

new glomeruli end in the final weeks of gestation and no new structures are formed postnatally. But when neonates are born premature their kidneys are not yet fully developed and in that respect their ongoing nephrogenesis is similar to the preterm rabbit pups. In our experiments we confirm that nephrogenesis is indeed still ongoing after birth in the both the preterm and term cohorts by the presence of a nephrogenic zone and changes in total glomerular count, kidney volume and weight (compared to the fetal kidneys at G28). At present we cannot perform long term experiments to study further kidney maturation in preterm rabbits as the pups fail to survive beyond 10 days when fed artificially. So we cannot determine if this delayed nephrogenesis after preterm birth catches up and results in structurally and functionally normal mature kidneys.

Next to the structural changes, we studied the functional differences between the two cohorts. It became clear that the kidneys of the preterm cohort suggest have a reduced functionality, shown by an increased urinary protein/creatinine ratio and higher levels of urinary protein. Different hypotheses can be put forward to explain an increased protein/creatinine ratio in preterm rabbits. A study by Stelloh et al. has shown that preterm birth in mice correlates with a decrease in nephron endowment and thus induces stress on the remaining glomeruli, leading to proteinuria and hyperfiltration [24]. In our data, however, there is no compensatory increase in glomerular size, which is typically seen along with hyperfiltration, arguing against this theory. An alternative explanation are potential structural differences within the glomerulus. Wharram et al. have previously shown in a transgenic rat strain – in which podocytes express the human diphtheria toxin receptor - that a <40% podocyte reduction resulted in transient proteinuria and a reversible reduction of renal function. In contrast, a >40% podocyte reduction was responsible for segmental sclerosis, sustained proteinuria and a reduction in renal function [25]. In addition, Tsukahara et al. have described increasing and sustained high levels of albuminuria with increasing degrees of prematurity in human preterm neonates, whilst in term human neonates the protein levels gradually decreased during the first 28 days of life~~. Thus,~~ demonstrating an increase in

glomerular permeability and a decrease in tubular resorption with increasing degrees of prematurity [26]. From this, we hypothesize that the increase in proteinuria in the absence of glomerular hypertrophy, found in preterm rabbits, may be caused by changes in the glomerular structure, such as podocyte immaturity or an “absolute” podocyte depletion (a decrease in the total number of healthy podocytes per glomerulus [27]).

This study is not without limitations. Firstly, an estimate of the total glomerular count (stereology based), was not obtained. We did, however, measure the glomerular density as an estimate for the amount of glomeruli per area. This measurement is thought to be susceptible to confounding factors such as section thickness and glomerular hypertrophy [27]. To minimize the effects of these possible confounders we have consistently used the same section thickness and measured the cross-sectional renal corpuscle surface area in all cohorts. Secondarily, kidneys were embedded in paraffin: a method known to cause 40-50% shrinkage of soft tissues such as the kidney [28]. Furthermore, the prophylactic use of amikacin antibiotics in both preterm and term groups could have altered our results through nephrotoxicity [29]. But, all experimental groups received these antibiotics in a dosage adjusted for bodyweight.

In conclusion, we have combined histology and functional parameters providing new insight in postnatal nephrogenesis in the preterm rabbit. We have shown that the preterm rabbit tends to linger in the immature glomerular stages compared to term. Importantly, preterm rabbits also show signs of proteinuria and a higher protein-to-creatinine ratio, suggestive of hyperfiltration which could in time lead to short- and long-term health consequences. Future research could focus on the processes that are involved in these changes and in the use of the preterm rabbit for pharmacokinetic studies.

Acknowledgements

JD, EL and JT conceived the study; DDW, TS and AG collected the data; DDW analyzed the data, performed statistical analyses and DDW and TS drafted the manuscript; JD, EL and JT gave important intellectual contribution and critically revised the final manuscript.

References

1. Black MJ, Sutherland MR, Gubhaju L. Effects of Preterm Birth on the Kidney. In: Manisha Manay, editors. Basic Nephrology and Acute Kidney Injury. Intech; 2012. pp 61-88.
2. Siewert-Delle A, Ljungman S. The impact of birth weight and gestational age on blood pressure in adult life: A population-based study of 49-year-old men. Am J Hypertens. 1998;11(8 I):946–53.
3. Keijzer-Veen MG, Dülger A, Dekker FW, Nauta J, van der Heijden BJ. Very preterm birth is a risk factor for increased systolic blood pressure at a young adult age. Pediatr Nephrol [Internet]. 2010;25(3):509–16.
4. Johansson S, Iliadou A, Bergvall N, Tuvemo T, Norman M, Cnattingius S. Risk of high blood pressure among young men increases with the degree of immaturity at birth. Circulation. 2005;112(22):3430–6.
5. Kistner A, Jacobson L, Jacobson SH, Svensson E, Hellstrom A. Low gestational age associated with abnormal retinal vascularization and increased blood pressure in adult women. Pediatr Res. 2002;51(6):675–80.
6. Bonamy AKE, Bendito A, Martin H, Andolf E, Sedin G, Norman M. Preterm birth contributes to increased vascular resistance and higher blood pressure in adolescent girls. Pediatr Res. 2005;58(5):845–9.
7. Dalziel SR, Parag V, Rodgers A, Harding JE. Cardiovascular risk factors at age 30 following pre-term birth. Int J Epidemiol. 2007;36(4):907–15.

8. Cooper R, Atherton K, Power C. Gestational age and risk factors for cardiovascular disease: Evidence from the 1958 British birth cohort followed to mid-life. Int J Epidemiol. 2009;38(1):235–44.
9. Kistner A, Celsi G, Vanpee M, Jacobson SH. Increased blood pressure but normal renal function in adult women born preterm. Pediatr Nephrol. 2000;15(3–4):215–20.
10. Salaets T, Gie A, Tack B, Deprest J, Toelen J. Modelling Bronchopulmonary Dysplasia in Animals: Arguments for the Preterm Rabbit Model. Curr Pharm Des. 2017;23(38):5887–901.
11. Bankhead P, Loughrey MB, Fernández JA, Dombrowski Y, McArt DG, Dunne PD, et al. QuPath: Open source software for digital pathology image analysis. Sci Rep [Internet]. 2017;7(1):16878.
12. Naruse K, Fujieda M, Miyazaki E, Hayashi Y, Toi M, Fukui T, et al. An immunohistochemical study of developing glomeruli in human fetal kidneys. Kidney Int. 2000;57(5):1836–46.
13. Thóny HC, Luethy CM, Zimmermann A, Laux-End R, Oetliker OH, Bianchetti MG. Histological features of glomerular immaturity in infants and small children with normal or altered tubular function. Eur J Pediatr. 1995;154(4 Supplement).
14. Sutherland MR, Gubhaju L, Moore L, Kent AL, Dahlstrom JE, Horne RSC, et al. Accelerated maturation and abnormal morphology in the preterm neonatal kidney. J Am Soc Nephrol. 2011;22:1365–74.
15. dos Santos AM, Fonseca Ferraz ML, Pinto Rodriguez ML, dos Reis MA, Miranda Corrêa RR, de Paula Antunes Teixeira V, et al. Assessment of renal maturity by assisted morphometry in autopsied fetuses. Early Hum Dev. 2006;82(11):709–13.
16. Pesce MA, Strande CS. A new micromethod for determination of protein in cerebrospinal fluid and urine. Clin Chem. 1973;19(11):1265–7.
17. Monteiro Carvalho Mori Da Cunha MG, Zia S, Arcolino FO, Carlon MS, Beckmann DV, Pippi NL, et al. Amniotic fluid derived stem cells with a renal progenitor

phenotype inhibit interstitial fibrosis in renal ischemia and reperfusion injury in rats. PLoS One. 2015;10(8).

18. Allegaert K, Van Den Anker JN. Clinical pharmacology in neonates: Small size, huge variability. In: Neonatology. 2014. p. 344–9.
19. Van Der Merwe J, Van Der Veeken L, Ferraris S, Gsell W, Himmelreich U, Toelen J, et al. Early neuropathological and neurobehavioral consequences of preterm birth in a rabbit model. Sci Rep. 2019;9(1):3506.
20. Gubhaju L, Sutherland MR, Yoder BA, Zulli A, Bertram JF, Black MJ. Is nephrogenesis affected by preterm birth? Studies in a non-human primate model. Am J Physiol Renal Physiol. 2009;297(6):F1668-77.
21. Huang HP, Tsai IJ, Lai YC, Cheng CH, Tsau YK. Early postnatal renal growth in premature infants. Nephrology. 2007;12(6):572–5.
22. Callaway DA, McGill-Vargas LL, Quinn A, Jordan JL, Winter LA, Anzueto D, et al. Prematurity disrupts glomeruli development, whereas prematurity and hyperglycemia lead to altered nephron maturation and increased oxidative stress in newborn baboons. Pediatr Res. 2018;83(3):702–11.
23. Fayez S, Sayed-Ahmed A, I Abo-Ghanema I, Elnasharty M. Morphogenesis of Rabbit Kidney Pre-and Postnatal. Vol. 41, Alexandria Journal of Veterinary Sciences. 2014. 35–49 p.
24. Stelloh C, Allen KP, Mattson DL, Lerch-Gaggl A, Reddy S, El-Meanawy A. Prematurity in mice leads to reduction in nephron number, hypertension, and proteinuria. Transl Res. 2012;159(2):80–9.
25. Wharram BL. Podocyte Depletion Causes Glomerulosclerosis: Diphtheria Toxin-Induced Podocyte Depletion in Rats Expressing Human Diphtheria Toxin Receptor Transgene. J Am Soc Nephrol [Internet]. 2005;16(10):2941–52.
26. Tsukahara H, Fujii Y, Tsuchida S, Hiraoka M, Morikawa K, Haruki S, et al. Renal handling of albumin and beta-2-microglobulin in neonates. Nephron. 1994;68(2):212–6.

27. Puelles VG, Bertram JF. Counting glomeruli and podocytes: Rationale and methodologies. Vol. 24, Current Opinion in Nephrology and Hypertension. 2015. p. 224–30.
28. Dorph-Petersen KA, Nyengaard JR, Gundersen HJG. Tissue shrinkage and unbiased stereological estimation of particle number and size. J Microsc. 2001;204(3):232–46.
29. Mingeot-Leclercq MP, Tulkens PM. Aminoglycosides: Nephrotoxicity. Antimicrob Agents Chemother. 1999;43(5):1003–1012.

Supporting information

S1 Fig. Illustrative overview of the histology methods performed. (A): Representative image of the nephrogenic zone as found in the outer renal cortex. The lower border of the nephrogenic zone is highlighted (white line). (B): Example histology slides for G28, G28 + 7 and G31 + 4. Provides a quick overview of the relative size. (C): Illustrative example of the analysis methodology. The kidney was divided in 8 sectors using *grid lines*. The upper or lower pole was randomly selected and all *glomerular cross-sections* were counted and staged. The *renal cortex surface* was measured by tracing just underneath the outer renal capsule and along the boundary between medulla and cortex. The *nephrogenic zone width* was measured in 8 sectors; the *renal corpuscle surface area* was measured in the 4 sectors in the randomly selected pole.

S1 Table. Baseline characteristics of units of analysis: G28, G28 + 7, and G31 + 4, presented as *mean* and *95% confidence intervals.*

Figure 1

Click here to access/download;Figure;Fig1.tif

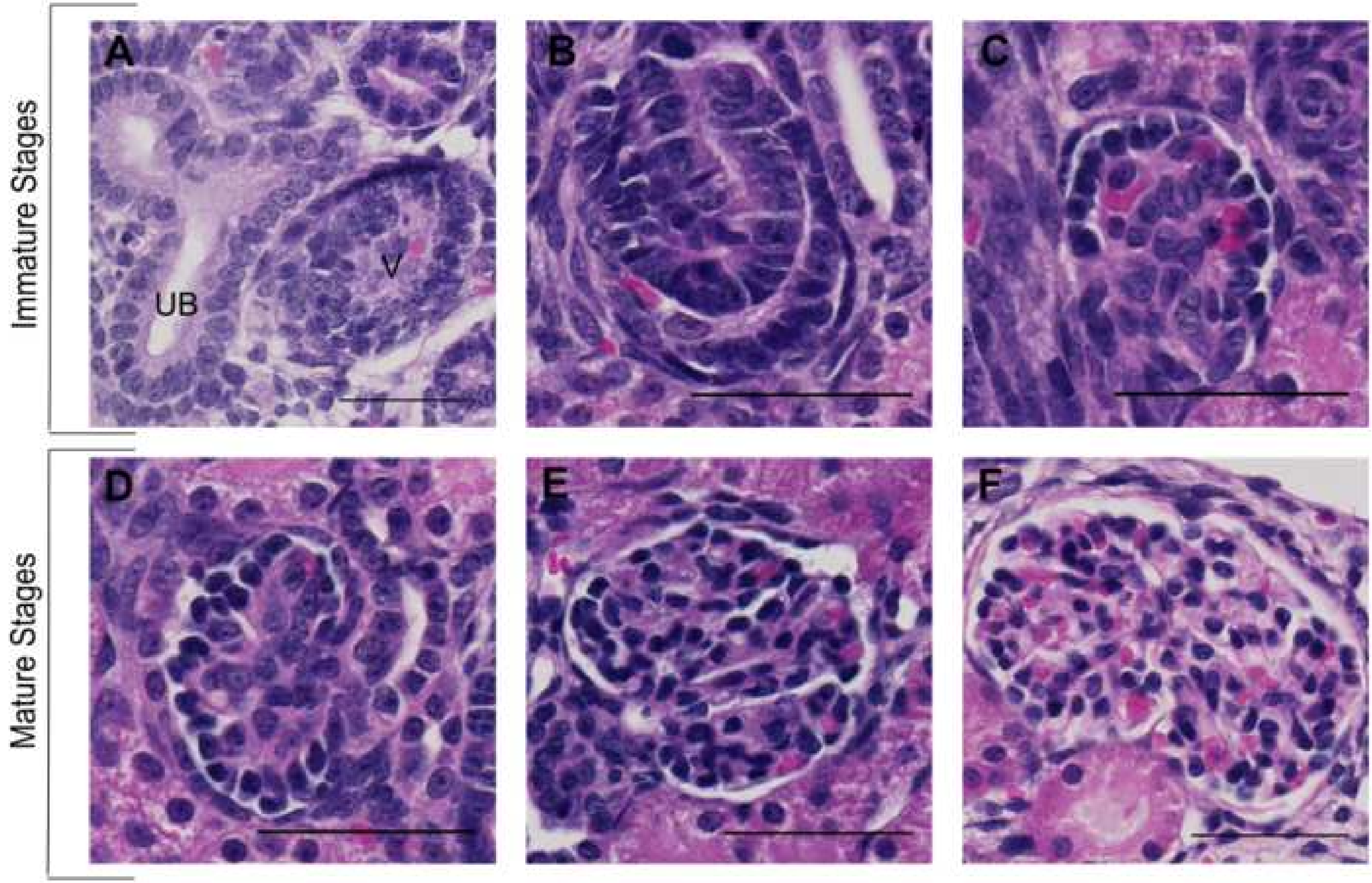

Figure 2 Click here to access/download;Figure;Fig2.tif

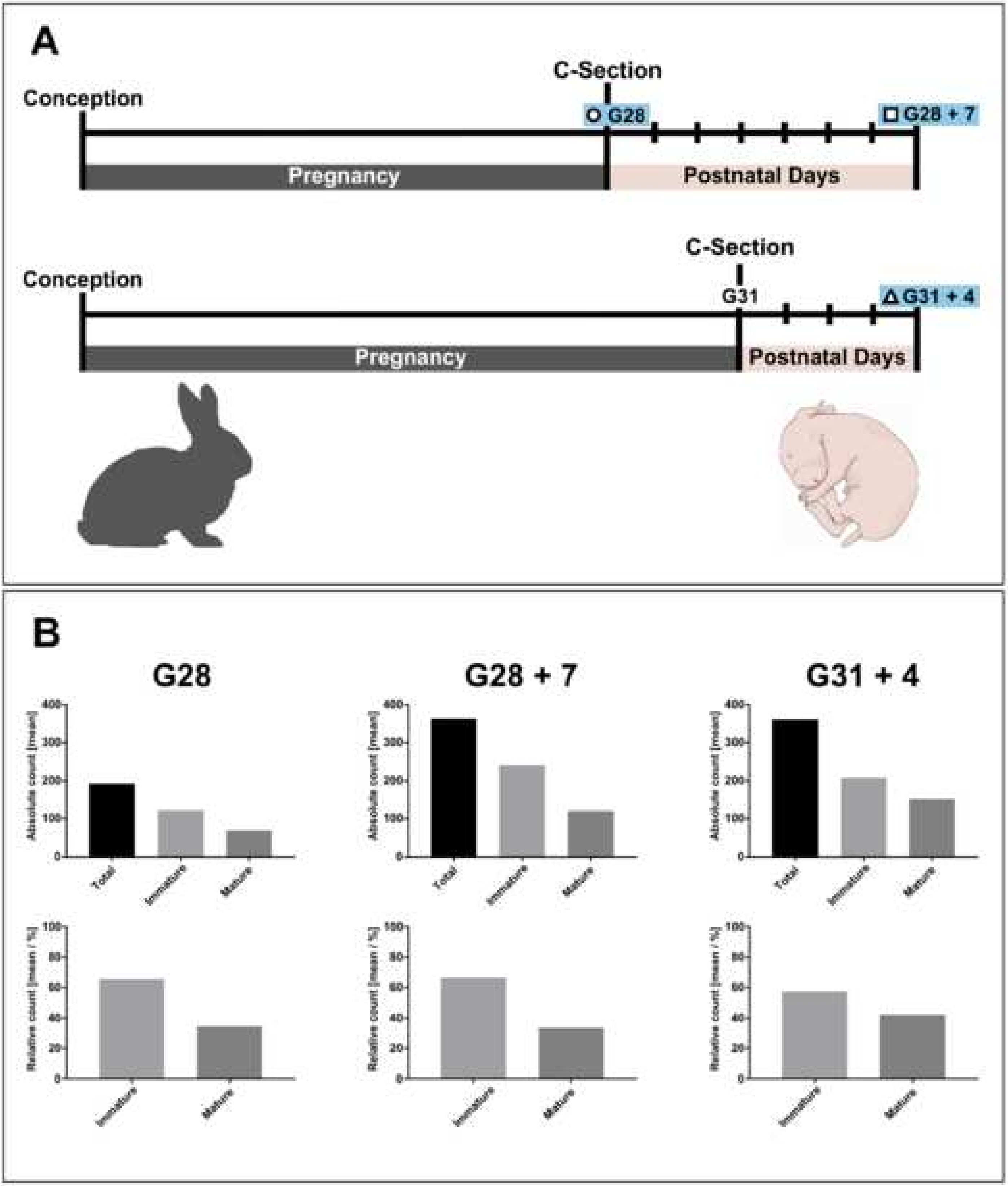

Figure 3 Click here to access/download;Figure;Fig3.tif

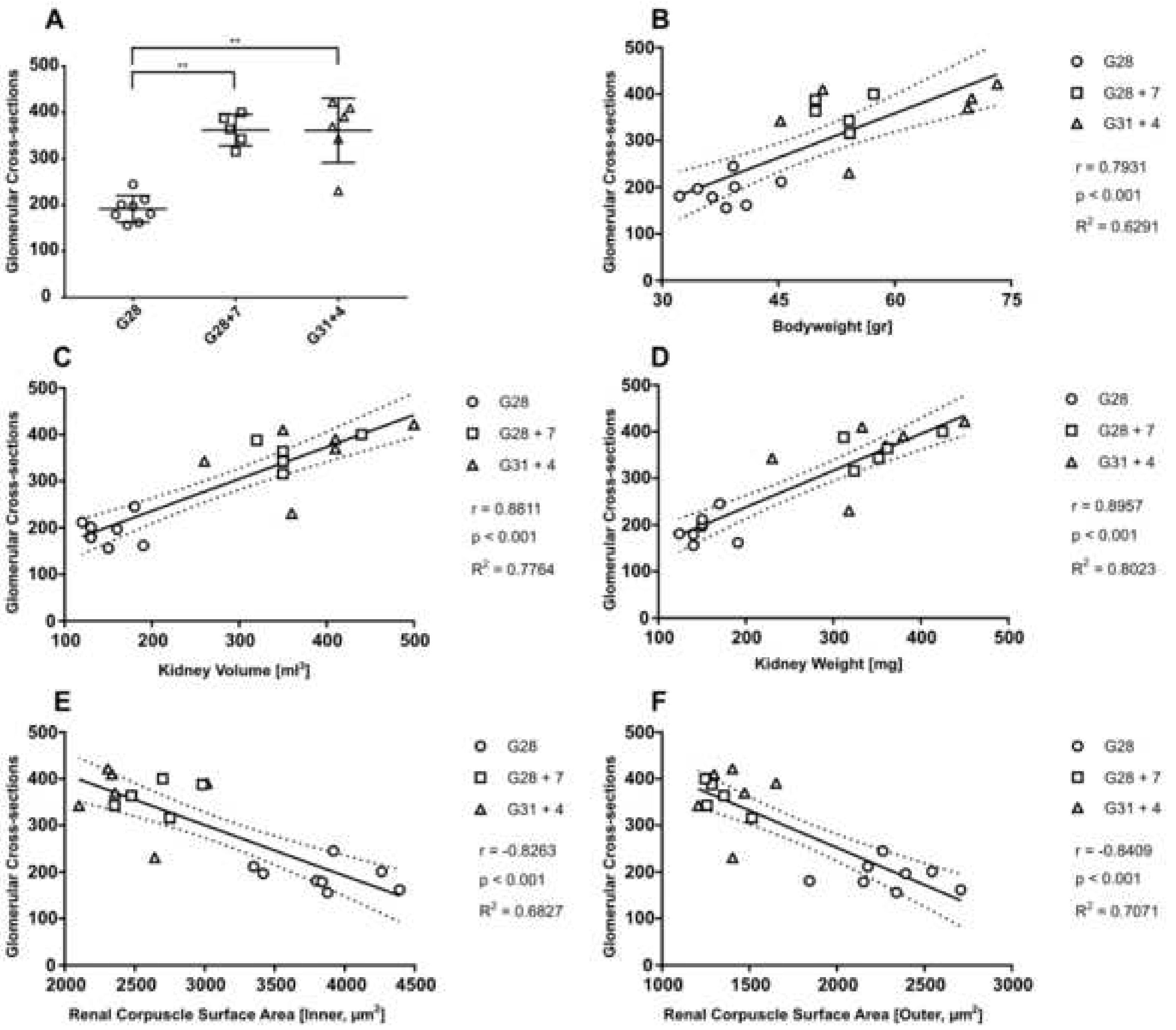

Figure 4

Click here to access/download;Figure;Fig4.tif

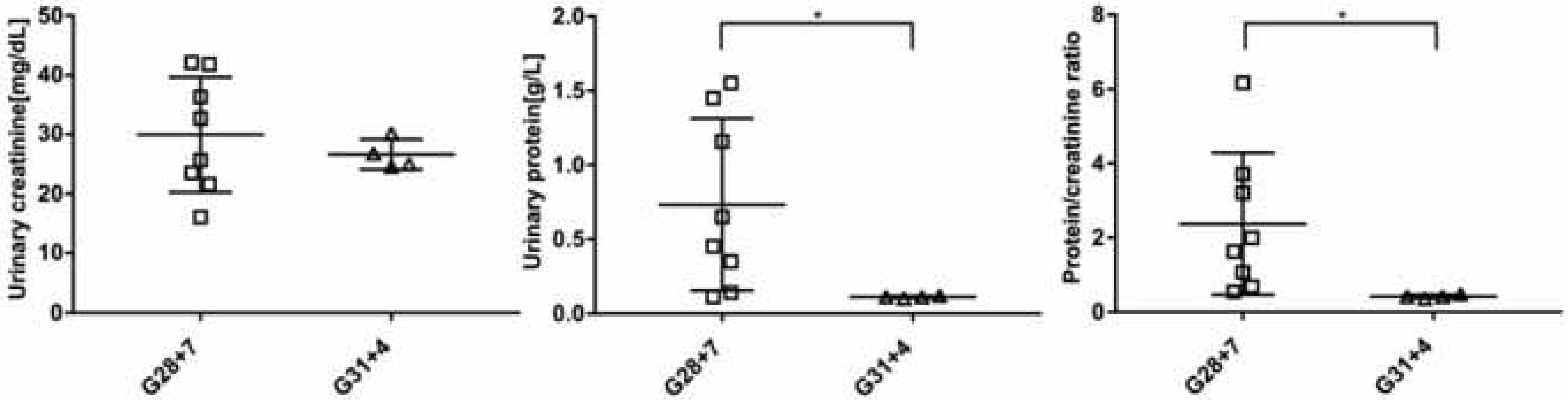

Supplementary figure 1

Click here to access/download
Supporting Information
S1_Fig.tif

Supplementary table 1

Click here to access/download
Supporting Information
S1_Table.pdf
